# Validation of QF-PCR for prenatal diagnoses in a Brazilian population

**DOI:** 10.6061/clinics/2017(07)02

**Published:** 2017-07

**Authors:** Renata Wendel de Moraes, Mario Henrique Burlacchini de Carvalho, Antonio Gomes de Amorim-Filho, Rossana Pulcineli Vieira Francisco, Renata Moscolini Romão, José Eduardo Levi, Marcelo Zugaib

**Affiliations:** I Departamento de Ginecologia e Obstetricia, Faculdade de Medicina FMUSP, Universidade de Sao Paulo, Sao Paulo, SP, BR; II Fundação Pró-Sangue, Hemocentro de São Paulo, SP, BR

**Keywords:** Prenatal Diagnosis, Aneuploidy, QF-PCR, Brazilian Population

## Abstract

**OBJECTIVES:**

Quantitative fluorescence polymerase chain reaction (QF-PCR) is a rapid and reliable method for screening aneuploidies, but in Brazil, it is not used in public services. We investigated the accuracy of QF-PCR for the prenatal recognition of common aneuploidies and compared these results with cytogenetic results in our laboratory.

**METHOD:**

A ChromoQuant QF-PCR kit containing 24 primer pairs targeting loci on chromosomes 21, 13, 18, X and Y was employed to identify aneuploidies of the referred chromosomes.

**RESULTS:**

A total of 162 amniotic fluid samples analyzed using multiplex QF-PCR were compared with karyotyping analysis. The QF-PCR results were consistent with the results of cytogenetic analysis in 95.4% of all samples.

**CONCLUSION:**

QF-PCR was demonstrated to be efficient and reliable for prenatal aneuploidy screening. This study suggests that QF-PCR can be used as a rapid diagnostic method. However, rearrangements and some mosaic samples cannot be detected with this test; thus, those exceptions must undergo cytogenetic analysis.

## INTRODUCTION

The most frequent chromosome abnormality identified in humans is aneuploidy, which occurs in 5% of all pregnancies and is the leading cause of pregnancy loss 1. Numerical and structural chromosome abnormalities are detected in approximately one in 200 newborns, and these abnormalities are the most common causes of developmental disabilities and congenital malformations in humans 2.

In most countries, cytogenetic analysis has become an important component of prenatal diagnosis. Since the origin of cytogenetics, karyotyping has been used as a gold standard test for aneuploidy diagnosis 3,4. The accuracy of this test is approximately 99%; however, this technique is laborious and involves a long reporting time because of the necessity for cell culture 2-5. For most clinical genetics laboratories, the results can take 10 to 15 days, which is a long waiting period for anxious parents.

To reduce parent stress and the time for diagnosis, the demand for rapid diagnostic methods that do not require cell culture has increased. These tests can detect aneuploidies using molecular techniques, such as quantitative fluorescent polymerase chain reaction (QF-PCR) 6. QF-PCR involves the amplification of small repetitive DNA sequences – short tandem repeats (STRs) – using fluorescent primers, followed by quantitative analysis of the products to evaluate the numbers of copies of specific chromosomes 7. Although a microsatellite marker is heterozygous, the ratio of its allele peak areas represents a disomic (1:1) or trisomic (2:1, 1:2, or 1:1:1) chromosome complement. A marker is uninformative if only a single peak is observed 8.

In 2000, QF-PCR was first introduced to the UK National Health Service as a validated and efficient diagnostic test 9. In Stockholm, since 2005, women have been able to choose between QF-PCR alone or full karyotype for prenatal diagnosis 10, and in the USA, since 2006, this method has been validated and available 11. In Brazil, this technique is not widely available: only a few private services offer either QF-PCR or fluorescence in situ hybridization (FISH) in association with cytogenetic culture; furthermore, none of them are offered as a routine public service.

The lack of studies involving prenatal diagnosis using QF-PCR methods in Brazil contributed to this study. The aims of this study were to investigate the diagnostic test performance of QF-PCR for the detection of trisomy 13, 18, and 21 and sex chromosome aneuploidies in high-risk pregnancies, to compare these results with karyotypes and to introduce this method as routine practice in our university hospital.

## MATERIALS AND METHODS

### Study samples

The data for this study were derived from the analysis of stored amniotic fluid obtained during prospective amniocentesis for prenatal diagnosis. A total of 162 samples of amniotic fluid were collected from pregnant women who were referred to the Obstetric Clinic at the Hospital das Clínicas da Faculdade de Medicina da Universidade de São Paulo (HC-FMUSP), São Paulo, Brazil, from August 2009 to May 2013. This study was approved by the University Ethics Committee (CAPPESq-0789/08) in accordance with the ethical standards of the responsible committee on human experimentation. All participants in the study provided written informed consent.

QF-PCR tests were performed by the same person (first author), and samples were analyzed with the karyotype results blinded. All women received genetic counseling, and routine informed consent was obtained in all cases included in the study. The clinical indications for karyotyping investigation included increased nuchal translucency (NT) thickness, previous child with chromosome abnormalities and structural fetal malformations. Most prenatal samples were collected between 12 and 34 weeks of gestation. At least 20 mL of amniotic fluid was collected from each pregnant woman, and 1 mL was allocated for our research and stored at – 80 °C for posterior analysis. Conventional cytogenetic analyses were performed on all the prenatal samples, which were cultured according to standard procedures in the HC-FMUSP, and results were issued between 14 and 21 days later.

### DNA extraction

DNA was extracted from fetal cells, which were obtained from amniotic fluid (1.0 mL) using a QIAamp DNA Blood Mini kit (Qiagen, Germany; CITOGEM Biotecnologia Ltda). Nucleic acids were eluted in a final volume of 65 μL of elution buffer. After extraction, the quality and quantity of DNA were evaluated by spectrophotometry on a NanoDrop 2000 (Thermo Fisher Scientific, USA).

### Markers used

The assay uses multiplex PCR targeting STR markers to assess the copy numbers of chromosomes 13, 18, 21, X and Y. A total of 24 markers were selected for this study: 5 markers for chromosome 13 (D13S797, D13S742, D13S634, D13S628, and D13S305), 6 for chromosome 18 (D18S391, D18S976, D18S819, D18S390, D18S386, and D18S535), 6 for chromosome 21 (D21S1409, D21S11, D21S1411, D21S1246, D21S1444, and D21S1435), 3 for chromosome X (DXS6854, DXS6803, and XHPRT), 1 STR on the Y chromosome (SRY) and 2 for regions on the X and Y chromosomes (DXYS218 and X22). The amelogenin gene (AMXY) and SRY were used to allow for the assessment of fetal sex. QF-PCR is a quantitative method: the areas and the heights of the peaks are comparable.

### Multiplex QF-PCR

PCR amplification was performed in two reactions using 10 µL of the extracted DNA and 15 µL of ChromoQuant QF-PCR v.3 (Cybergene AB, Sweden) mix. After initial denaturation at 95 °C for 15 min, 26 cycles of amplification were performed (denaturation at 94 °C for 30 s, annealing at 57 °C for 1 min and extension at 71 °C for 2 min) followed by final extension at 71 °C for 5 min and 60 °C for 1 hour. The reaction was performed in a thermal cycler (Eppendorf, Germany).

### Genescan analysis and reporting

The amplified samples were pooled together (1.0 μL) with 12 μL of Hi-Di formamide (Applied Biosystems, USA) and 0.3 μL of ROX500 (Applied Biosystems, USA). This mixture was denatured at 95 °C for 3 min, transferred to ice and left until the sample was loaded on an ABI 3130 Genetic Analyzer. The samples were run on a POP7 polymer and a 36-cm capillary, and the obtained results were analyzed with Gene Mapper V4.0 (Softgenetics, USA). For reporting, the peak height ratio or area ratio was calculated. We considered a minimum of two markers to be informative for reporting, following the manufacturer’s protocol. The allele ratio for the normal range was from 0.8 to 1.4. If the ratio ranged from 1.8 to 2.4 or 0.45 to 0.65, it was considered trisomy positive for three individual peaks (1:1:1) with respect to each marker considered trisomy positive.

### Cytogenetic analysis

Amniocytes were cultured and G banding was performed for all cases. These samples were analyzed at the cytogenetic laboratory at the Hospital das Clínicas da Universidade de São Paulo. Routine evaluation of each case involved the analysis of 20 random metaphase spreads from two independent cultures. Karyotypes were described according to the International System for Human Cytogenetic Nomenclature (ISCN2013).

### Statistical analysis

To determine appropriate sample size, a power calculation using a sensitivity of 83.73% was performed as described by Rostami et al. 12 in a group of pregnant women selected for karyotype studies according to increased maternal age and positive screen test; the prevalence of an abnormal karyotype was 4%. Considering that 55% of the samples in the present study presented abnormal karyotypes with a maximum estimated error of 10%, the 95% confidence interval would be achieved in a sample size of 95 cases.

## RESULTS

### Patient demographics

The samples obtained from the Brazilian population included 105 white volunteers, 45 brown volunteers, 9 black volunteers and 2 indigenous volunteers; 1 was unknown. Classifications of ethnicity were made according to the self-assessment of the volunteers.

The mean maternal age was 29 years old (range, 14-49 years); 55 women (33.95%) were 35 years of age or older. The median gestational age of amniocentesis was 23 weeks (range: 11 weeks to 34 weeks).

The results of 162 QF-PCR samples were compared to results obtained by culture conventional cytogenetic analysis, as presented in Table 1. There were two false-positives and one false-negative. The false-negative QF-PCR result was observed in one mosaicism for Turner syndrome; this sample was analyzed and reported as a normal female fetus. False-positives were found in 2 cases: one normal male with trisomy 18 and one normal female with polyploidy metaphases that showed abnormal results for chromosomes 21, 18, 13 and X. There were 151 samples corresponding with cytogenetic results (98.05%), excluding cases with chromosomal rearrangement. Table 2 shows the sensitivity, specificity, and positive and negative predictive values for all cases excluding those with rearrangements and mosaicism. The number and percentage of aneuploidies detected in fetuses were compared with different ages of the mothers, as shown in Table 3.

Among the discrepant results, 8 were due to chromosomal rearrangements, including one addition (46, XX, +add (7) (q33)), two translocations (46, XX, +13 rob (13, 14) (q10q10), 46, Y, t (X,14) (q22q31)), three inversions (46, XX, +i (1) (q10), 46, XY inv (9), 46, XY, inv (9) (p12q13)), one deletion (46, XX, del (11) (q22)), and one duplication (46, XY, dup (3)(p21.2p25)). In five cases of mosaicism, QF-PCR identified four abnormal trisomy karyotypes of the corresponding chromosomes (Table 4).

### Analysis of the heterozygosities of STR markers

The heterozygosities of selected markers for QF-PCR are shown in Table 5. The markers D21S1435, D21S1409, and D21S1246 more commonly showed in a triallelic pattern, whereas the markers D13S742, D21S11, and D18S386 showed a diallelic pattern. For the sex chromosomes, the marker X22 had the highest frequency of heterozygosity.

## DISCUSSION

In recent years, QF-PCR for the detection of common chromosomal trisomies has been introduced as a validated method at a number of cytogenetic centers 13-16. Our study presents results based on the application of QF-PCR for the rapid detection of aneuploidies in chromosomes 13, 18, 21, X and Y on 162 amniotic fluid samples in the Brazilian population.

The QF-PCR results alone were in accordance with 98.05% of all karyotypes excluding cases with chromosomal rearrangements. These results are compatible with other recent reports in the literature. Lildballe et al. 17 analyzed 2,550 samples from chorionic villus sampling (CVS) and amniotic fluid from high-risk pregnancies and reported positive and negative predictive values greater than 99.8%. In this work, different predictive values were reported for each chromosomal abnormality, and even for mosaic trisomies, the detection rate was higher than 99.8%. Rostami et al. 12 reported 4,058 samples analyzed for QR-PCR with a detection rate of 98.59%. Tekcan et al. 18 compared 100 amniotic fluid samples with karyotype results and obtained 99% concordance on 100 samples, including 4 abnormalities. These three authors reported higher detection rates than in our study; however, the percentages of total chromosomal abnormalities in these studies were 7.6% and 4.1%, respectively, whereas in the current study, approximately half of the population was abnormal. This difference in the prevalence of aneuploidy could be explained by the indication of the karyotype that was primarily due to fetal malformation in our study. Łaczmańska et al. 19 analyzed 100 samples of amniotic fluid and obtained compatibility in 95 cases (95%), which agrees with our study due to the higher number of chromosomal abnormalities, which were found in 28 pregnancies (29.5%).

Our detection rate of 98.8% included five cases of mosaicisms; among which four of these samples returned abnormal QF-PCR results, which was in agreement with the chromosome involved in the abnormality. If we consider that an abnormal result should be further confirmed by cytogenetic analysis, these four cases could be considered positive screen tests for aneuploidy in QF-PCR. Additionally, one Turner mosaic sample was revealed to be normal in QF-PCR testing. This result is in accordance with the literature and can be explained by lower rates of mosaic cells (less than 15%) 19-22.

Of the two false-positive results, one revealed a triploid female upon QF-PCR with a normal polyploidy metaphases karyotype upon cytogenetic analysis. These polyploidy metaphases might be the reason for the false-positive result, though its significance is unknown. A second false-positive was a normal male, which was revealed as trisomy 18. The sample was bloodstained, so this false result might be explained by maternal contamination.

The markers D21S1435, D13S742, D13S797, and D18S1386 were the most frequently informative in all cases of trisomies 21, 13 and 18, respectively, in our population, displaying a higher frequency of heterozygosity.

QF-PCR is a rapid, robust and accurate diagnostic method for detecting common aneuploidies in high-risk pregnancies, with results available in two days because fetal cells do not have to be cultured.

The Implementation of QF-PCR at a public referral center or laboratory could improve patient care and reduce overall health costs. A strategy could be proposed in which, in combination with traditional 1^st^ trimester screening tests, pregnant women could be referred for karyotyping via QF-PCR due to advanced maternal age, anxiety, NT <3 mm or a high-risk for trisomies; this would eliminate the need for karyotyping by culture. Karyotyping still should be applied in pregnancies with normal QF-PCR results and the presence of either structural abnormalities detected by ultrasound or two or more soft markers for Down syndrome as well as a family history of chromosome rearrangement.

This approach has been implemented in London and has led to a 99.9% detection rate of any chromosomal abnormality, which has reduced the need for a full karyotype analysis to only 25% of pregnancies 23. For the public health system in Brazil, where most pregnant women do not have access to fetal karyotyping, an initial investigation with a rapid and feasible assay such as QF-PCR would provide the opportunity to offer tests at a central laboratory and include more patients. Although QF-PCR cannot detect all chromosomal abnormalities, this assay can cover more than 97% of the predicted chromosomal anomalies when a fetal karyotype is requested.

As for any other prenatal test, employing this assessment requires investment in infrastructure, equipment and training for the lab staff. This test can decrease the demand for conventional cytogenetic analysis and can reduce parental anxiety as well as expand the reach of this test throughout the population.

## AUTHOR CONTRIBUTIONS

Moraes RW was responsible for the data collection, analysis and interpretation and manuscript drafting. de Carvalho MH was responsible for the study conception and design, manuscript drafting, critical review of the manuscript for intellectual content, approval of the final version of the manuscript, supervision of all aspects of the project. De Amorim-Filho AG was responsible for the data collection, analysis and interpretation, critical review of the manuscript for intellectual content. Francisco RP was responsible for the critical review of the manuscript for intellectual content, approval of the final version of the manuscript for submission. Romão RM was responsible for the data collection. Levi JE was responsible for the study conception and design, manuscript drafting, critical review of the manuscript for intellectual content, approval of the final version of the manuscript for submission, supervision of all aspects of the project. Zugaib M was responsible for the approval of the final version of the manuscript for submission.

## Figures and Tables

**Table 1:** Comparison of QF-PCR and conventional cytogenesis results in AF samples.

Karyotype	Cytogenetic, N (%)	QF-PCR, N (%)
**46, XX; 46, XY**	72 (44)	70 (43)
**47, XX +21; 47, XY +21**	28 (17)	28 (17)
**47, XX +18; 47, XY +18**	21 (13)	21 (13)
**47, XX +13; 47, XY +13**	9 (6)	9 (6)
**Turner syndrome (45, X)**	17 (10)	17 (10)
**Triploidy (69, XXX; 69, XXY)**	2 (1)	2 (1)
**Mosaics**	5 (3)	4 (2)
**Rearrangements**	8 (5)	0 (0)
**Total abnormalities**	90 (56)	81 (50)
**Test accuracy (%)**	100	93

**Table 2:** Statistical analysis of AF samples tested using QF-PCR in high-risk pregnancies with an aneuploid fetus.

Statistical analysis	AF samples without rearrangements cases (n=154) (%)	AF samples without mosaicism cases (n=157) (%)
**Sensitivity**	98.78	90.59
**Specificity**	97.22	97.22
**Positive predictive value**	97.59	97.44
**Negative predictive value**	98.59	89.74
**Accuracy**	98.05	93.63

**Table 3:** Number and percentage of aneuploidies in fetuses detected using QF-PCR as stratified by the mother’s age.

Mother’s age	Total Aneuploidies N (%)	47, XX/XY +21	47, XX/XY +18	47, XX/XY +13	45, X	69, XXX, 69, XXY
<35	48 (63)	13 (17)	14 (18)	7 (9)	12 (16)	2 (3)
≥35	29 (37)	15 (19)	7 (9)	2 (3)	5 (6)	0 (0)
**Total**	77 (100)	28 (36)	21 (27)	9 (12)	17 (22)	2 (3)

**Table 4:** Discrepant cases of QF-PCR.

Rearrangements	QF-PCR
**46, XX, +add (7) (q33)**	46, XX
**46, XX, +13 rob (13, 14) (q10q10)**	46, XX
**46, Y, t (X,14) (q22q31)**	46, XY
**46, XX, +i (1) (q10)**	46, XX
**46, XY inv (9)**	46, XY
**46, XY, inv (9) (p12q13)**	46, XY
**46, XX, del (11) (q22)**	46, XX
**46, XY, dup (3)(p21.2p25)**	46, XY

Mosaics	QF-PCR
**45, X0 / 46, XX, +mar**	46, XX
**46, XY / 47, XY, +18**	47, XY, +18
**46, XY / 47, XY, +18**	47, XY, +18
**47, XX, +13 / 48, XX, +13 mar**	47, XX, +13
**47, XX, +mar / 47, XX, +13**	47, XX, +13

**Table 5:** QF-PCR results for each STR marker of chromosomes 13, 18, 21, X and Y.

STR	Chromosome location	Mono allelic, N (%)	Diallelic, N (%)	Triallelic, N (%)
**AMEL**	Xp22.31 – Xp22.1Yp11.2	93 (58)	66 (41)	2 (1)
**DXYS218**	Xp22.32 / Yp11.3	67 (41)	91 (56)	4 (2)
**DXS6803**	Xq21.31	100 (62)	58 (36)	3 (2)
**DXS6854**	Xq26.1	107 (66)	52 (32)	3 (2)
**XHPRT**	Xq26.1	97 (60)	63 (39)	2 (1)
**X22**	Xq28Yq	38 (66)	10 (17)	10 (17)
**D21S11**	21q21.1	26 (44)	11 (19)	22 (37)
**D21S1246**	21q22.2	40 (25)	97 (60)	25 (15)
**D21S1409**	21q21.2	48 (30)	86 (53)	27 (17)
**D21S1411**	21q22.3	25 (42)	11 (19)	23 (39)
**D21S1435**	21q21.1	36 (23)	96 (60)	28 (18)
**D21S1444**	21q22.13	33 (49)	10 (15)	25 (37)
**D18S386**	18q22.1	22 (37)	11 (19)	26 (44)
**D18S390**	18q22.3-18q23	72 (44)	80 (49)	10 (6)
**D18S391**	18p11.31	39 (58)	10 (15)	18 (27)
**D18S535**	18q12.3	31 (53)	10 (17)	18 (31)
**D18S819**	18q11.2	39 (60)	10 (15)	16 (25)
**D18S976**	18p11.31	41 (57)	10 (14)	21 (29)
**D13S305**	13q13.3	57 (38)	80 (54)	12 (8)
**D13S628**	13q31.1	45 (71)	10 (16)	8 (13)
**D13S634**	13q21.33	50 (32)	96 (61)	12 (8)
**D13S742**	13q12.13	14 (35)	13 (33)	13 (33)
**D13S797**	13q33.2	36 (60)	11 (18)	13 (22)
